# Ultrathin acoustic cloaking by a conformal hybrid metasurface

**DOI:** 10.1038/s41598-019-49148-3

**Published:** 2019-09-03

**Authors:** Yihe Wang, Ying Cheng, Xiaojun Liu

**Affiliations:** 1 Key Laboratory of Modern Acoustics, Department of Physics and Collaborative Innovation Center of Advanced Microstructures, Nanjing University, Nanjing, 210093 China; 2 State Key Laboratory of Acoustics, Institute of Acoustics, Chinese Academy of Sciences, Beijing, 100190 China

**Keywords:** Acoustics, Metamaterials

## Abstract

Ultrathin acoustic cloaking of obstacles with arbitrary shape is achieved by a conformal hybrid metasurface, which is composed of an outer layer of phase-control metasurface (PCM) and an inner layer of near-zero-index metasurface (NZIM). Here, the PCM and NZIM are discretized into two types of labyrinth elements. The NZIM is functionally equivalent to an equiphase area and can guide the waves around the obstacle, while the PCM can perpendicularly transfer the incident waves to the NZIM and then control the emergent waves from NZIM to propagate along the original incident direction. The efficient cloaking by hybrid metasurface tightly covered on the edges of the square and circular obstacles is demonstrated, with a total thickness only 0.62 times of operating wavelength.

## Introduction

Acoustic cloaking refers to hiding the features of obstacle by guiding waves around the it, and making the obstacle undetectable consequently. The main methods of acoustic cloaking are divided into two categories, i.e., the reflection-type^1–8^ and the transmission-type^9–20^. The reflection-type acoustic cloaking, which is so-called the ground cloak or the carpet cloak, is hiding the obstacle by a reflecting surface. The reflecting surface locates above the obstacle and can provide reflected waves just like the incident waves reflecting from a plane mirror. Recently, an arc-shape carpet cloak achieved by a thin shell-type acoustic metasurface is demonstrated, which provides the possibility for the carpet acoustic cloak with arbitrary shapes^8^. On the other hand, the transmission-type acoustic cloaking has been verified, both theoretically and experimentally, based on the theory of transformation acoustics, which employ the coordinate transformation^9–11,17–20^ as typical methodology and inhomogeneous anisotropic metamaterials as the cloaking shell. In addition, the near-zero-index metamaterials^13–15^ and metacages^16^ pave another way to achieve transmission-type cloaking. However, the thickness of such transmission-type cloak is much larger than the incident wavelength. When cloaking an obstacle with irregular shape, the shell thickness should be even bigger, which greatly limits the potential applications.

Recently, an optical cloaking mechanism is proposed based on the transparent metasurface^21–28^ and zero-index materials^29,30^. The transparent metasurface, which wraps around the zero-index materials, can manipulate the wavefront of incoming or outgoing waves. The zero-index materials can guide the perpendicular incident waves around the obstacle and cause the waves to exit vertically. Thus, the outgoing waves can be controlled to propagate along the original incident direction and make the obstacle undetectable. Inspired by this principle, in this paper, we extend it to acoustic cloaking, and hybridize the phase-control metasurface (PCM) with a high transmittance and a near-zero-index metasurface (NZIM) with a near-zero density to realize the ultrathin acoustic cloaking. Two kinds of labyrinth structures are used as the PCM and NZIM, respectively. It is found that the hybrid metasurface (HM) with a thickness only 0.62 times of work wavelength can hide the features of obstacle with various shapes.

## Results

### Theoretical model of conformal HM

**Figure 1:**
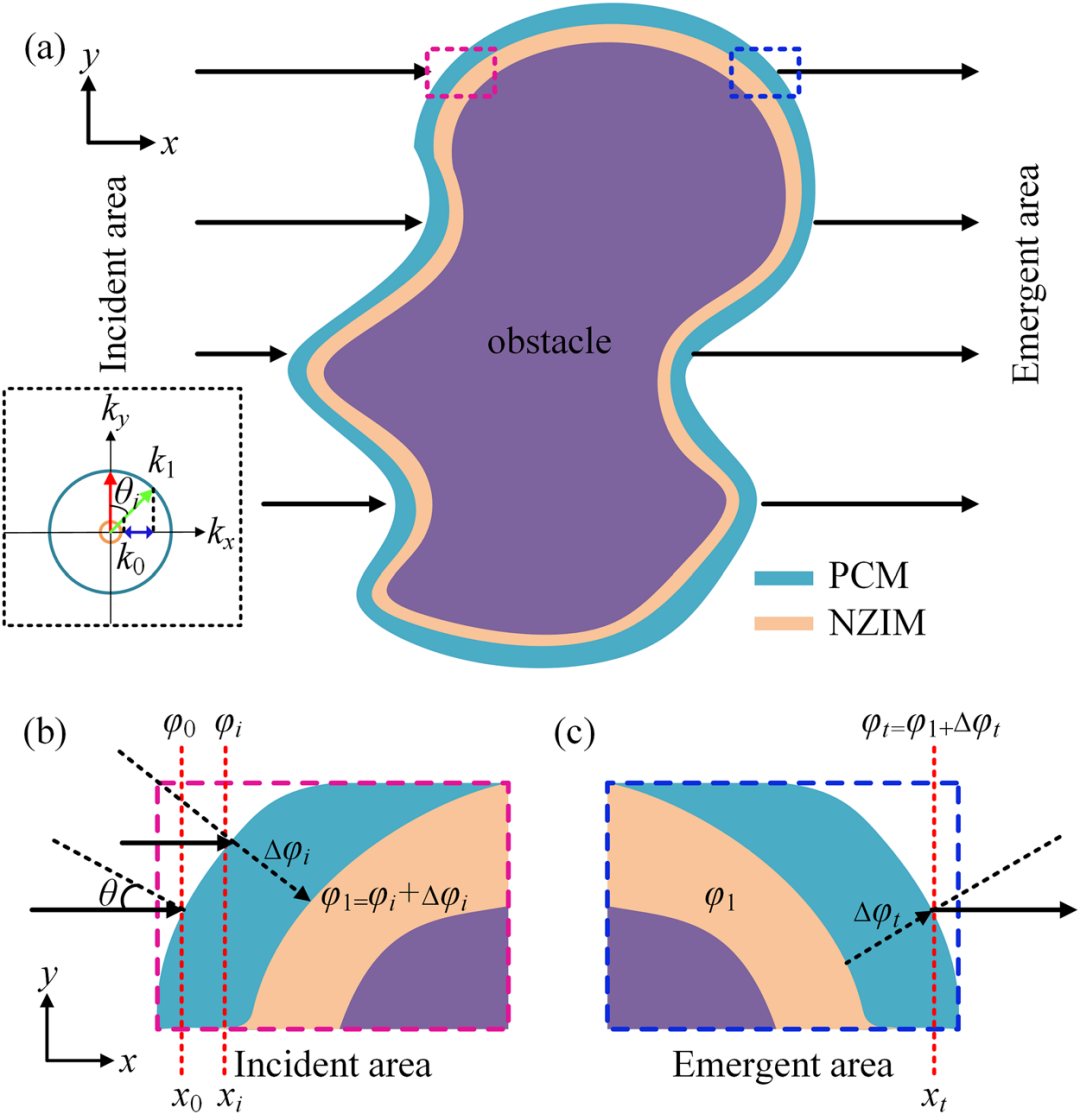
(**a**) Schematic diagram of acoustic hybrid metasurface. From inside out: obstacle with arbitrary shape, near-zero-index metasurface (NZIM) and phase-control metasurface (PCM). The inset in black dashed box shows the momentum space. The radii of blue and dusty pink circle are *k*_1_ and *k*_0_, respectively. The red (green) arrow indicates the normal incidence (oblique) incidence, and the blue arrow represents the transverse momentum mismatches when *θ*_*i*_ > *θ*_*c*_ **(b**,**c)** Zoom-in view of the incident area at the pink-outlined locations and the emergent area of the blue-outlined area indicated in **(a)**, respectively. *θ* represents the incident angle on PCM. *φ*_0_, *φ*_*i*_ and *φ*_*t*_ are the initial phase of the waves in the air at the positions *x*_0_, *x*_*i*_ and *x*_*t*_, respectively. *φ*_1_ is the phase of waves in the NZIM and stay the same at different positions in NZIM. Δ*φ*_*i*_ and Δ*φ*_*t*_ are the phase shift induced by PCM in incident area and emergent area, respectively.

As schematically illustrated in Fig. 1(a), the conformal HM consists of two layers, which wraps around an irregular obstacle. The outer layer is the PCM and the layer adjacent to the obstacle with arbitrary shape is the NZIM. For illustration, we assume that the incident plane waves propagate along the *x* direction. The PCM can provide different phase shifts for incident waves at different positions. The phase shifts induced by PCM at different positions are related to the properties of NZIM. Thus, we should first characterize the properties of NZIM. When plane waves are incident onto the NZIM from PCM, the critical angle at total reflection is ${\theta }_{c}=si{n}^{-1}(\frac{{n}_{0}}{{n}_{1}})$. Here, *n*_0_ and *n*_1_ represent the refractive index of the NZIM and PCM, respectively. Thus, the critical angle is close to zero for ${n}_{0}\approx 0$. In the momentum space, as shown in the inset of Fig. 1(a), the red and green arrow represent the normal incidence and oblique incidence, respectively. By matching the parallel component of the incident wavevector *k*_1_ on to the equifrequency curve of NZIM, one is able to find the propagable direction of the refracted beam. Since ${k}_{1}\gg {k}_{0}\approx 0$, the transverse momentum mismatches when the incident angle *θ*_*i*_ is greater than the critical angle *θ*_*c*_ (${\theta }_{i} > {\theta }_{c}\approx {0}^{\circ }$), resulting in the inability to pass through NZIM. That is to say, the oblique incident waves will be totally reflected. It is obvious that the phase variation of waves propagating in the NZIM is very small which can be regarded as an equiphase surface. Then the NZIM can guide the waves around the obstacle and the transmitted waves are also emitted vertically along the surface of NZIM. Based on the ‘tunneling effect’^31^, the normal incident waves can tunnel through the NZIM layer with a low reflectance and high transmittance. According to the above properties of NZIM, the phase profile of PCM can be designed. In order to achieve cloaking, the waves passing through PCM should vertically incident onto the NZIM in the incident area, as shown in Fig. 1(b). In the incident area, the waves arriving at the PCM at different positions *x*_*i*_ have different initial phases *φ*_i_. To make sure that the waves passing through PCM are perpendicular to the NZIM, the PCM must provide corresponding phase shift Δ*φ*_*i*_ in different position *x*_*i*_. Then the phase of waves in the output end of PCM should be ${\phi }_{1}={\phi }_{i}+{\rm{\Delta }}{\phi }_{i}$. Here, *φ*_1_ is the phase of waves propagating in the NZIM, which can be any constant. The relation between the phase shift Δ*φ*_*i*_ and the position *x*_*i*_ can be expressed as

1 $$\begin{array}{lll}{e}^{-i{\phi }_{0}}{e}^{-i{k}_{0}({x}_{i}-{x}_{0})}{e}^{-i{\rm{\Delta }}{\phi }_{i}}={e}^{-i{\phi }_{1}} & {\rm{or}} & {\rm{\Delta }}{\phi }_{i}=-{k}_{0}{x}_{i}+{\phi }_{1}\end{array}.$$

Here, ${\phi }_{0}(={k}_{0}{x}_{0})$ is the initial phase of incident waves at the position *x*_0,_ *k*_0_ is the wave vector in free space. As shown in Fig. 1(c), the emitted waves from NZIM should still travel in the original direction after the PCM. In the emergent area, the phase of waves propagating in the NZIM remains the same *φ*_1_. In order to achieve cloaking, the phase of waves passing through the PCM should be equal to the phase of waves propagating in free space without any obstacle at the same position *x*_*t*_. The relation between the phase shift Δ*φ*_*t*_ and *x*_*t*_ can be expressed as

2 $$\begin{array}{lll}{e}^{-i{\phi }_{1}}{e}^{-i{\rm{\Delta }}{\phi }_{t}}={e}^{-i{k}_{0}({x}_{t}-{x}_{0})} & {\rm{or}} & {\rm{\Delta }}{\phi }_{t}={k}_{0}{x}_{t}-{\phi }_{0}-{\phi }_{1}\end{array}.$$

Thus, the phase profile of PCM can be obtained. By the combination of PCM and NZIM, the transmission-type hybrid cloaking metasurface for obstacle with arbitrary shape can be achieved.

### Effective parameter model of HM to achieve cloaking

**Figure 2:**
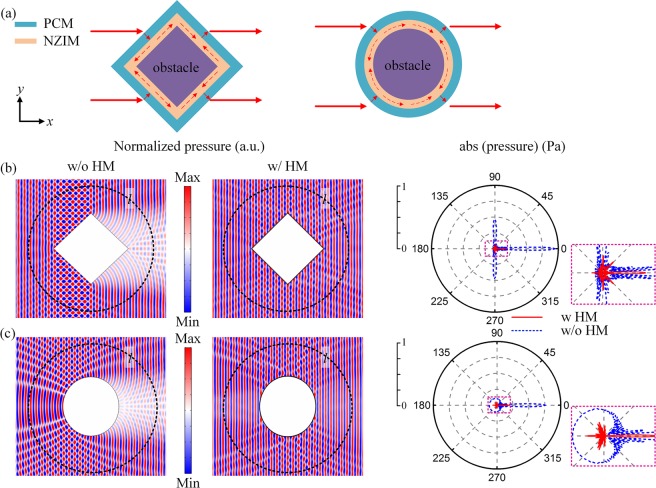
(**a**) Effective parameter model of the cloaking for square and circular obstacle, respectively. The *red* arrows denote the trace of the waves, which are guided around the obstacles in the NZIM layer. **(b)** Pressure field distributions and far-field scattering patterns of the uncloaked square obstacle without the HM and cloaked square obstacle with the HM. **(c)** Same as **(b)** but for the circular obstacle at *f* = 2395 Hz. Dashed circle *l* is the integration path when calculating the total scattering intensity.

Figure 2(a) shows the effective parameter model of the HM. Air acts as the background medium (*ρ*_air_ = 1.21 kg/m^3^, *c*_air_ = 343 m/s). An effective material with *n* = 0.001, *ρ* = 0.001 kg/m^3^ and *c* = 1000*c*_air_ is used as the NZIM. The outer layer is an effective anisotropic material whose acoustic impedance matches the acoustic impedance of air and can provide a -*π*~π phase shift as required by Eqs (1) and (2). For simplicity, *φ*_0_ and *φ*_1_ can be set as 0. Then, Eqs (1) and (2) can be expressed as ${\rm{\Delta }}{\phi }_{i}=-{k}_{0}{x}_{i}$ and ${\rm{\Delta }}{\phi }_{t}={k}_{0}{x}_{t}$, respectively. Figure 2(b) shows the simulated distributions of acoustic pressure fields and far-field scattering patterns of the square obstacle without and with the HM. The incident sound waves are excited by the background pressure field at *f* = 2400 Hz and propagate along the positive *x* direction. The length of the square obstacle is about 8.7 *λ* and the thickness of the HM is about 0.08 *λ*. Here, *λ* represents the operating wavelength at *f* = 2400 Hz. It can be seen from the contrast between pressure fields without or with the HM that, a good cloaking can be achieved with the HM. From the far-field scattering pattern of uncloaked rigid square obstacle without the HM, primary lobes exist before the square obstacle (0°) and perpendicular to the square obstacle (90° and 270°) which correspond to the sharp forward shadow and strong lateral reflections, respectively (blue dashed curve in right panel of Fig. 2(b)). When the rigid square obstacle is shielded by the HM metasurface, the scattered acoustic wave is dramatically reduced in almost all directions (see red solid curve in right panel of Fig. 2(b)). We can further analyze the cloaking effect of the HM quantitatively by calculating the scattering intensity. If no scatter exists in the sound field, the scattering intensity integral along the circle (the radius of circle *l* is about 11 *λ*) approximately equals to zero. If the square obstacle exists in the sound field only, the scattering intensity becomes 3.77 W/m^2^. When the obstacle is wrapped by the effective HM, the scattering intensity decreases to 0.47 W/m^2^. Thus, the HM can reduce the scattering intensity by 87.5% for the square obstacle. Similarly, Fig. 2(c) shows the distribution of acoustic pressure fields and far-field scattering patterns of the circle obstacle without and with the HM. The incident sound waves are excited by the background pressure field at *f*_1_ = 2395 Hz and propagate along the positive *x* direction. The radius of the circle obstacle is about 5.2 *λ*_1_ and the thickness of HM is about 0.08 *λ*_1_. Here, *λ*_1_ represents the wavelength at *f*_1_ = 2395 Hz. The scattering intensity integral along the circle decreases from 2.95 W/m^2^ without HM to 0.50 W/m^2^ with HM, indicating that the scattering intensity can be reduced by 83.1% for the circle obstacle.

### Construction of PCM unit and NZIM unit

**Figure 3:**
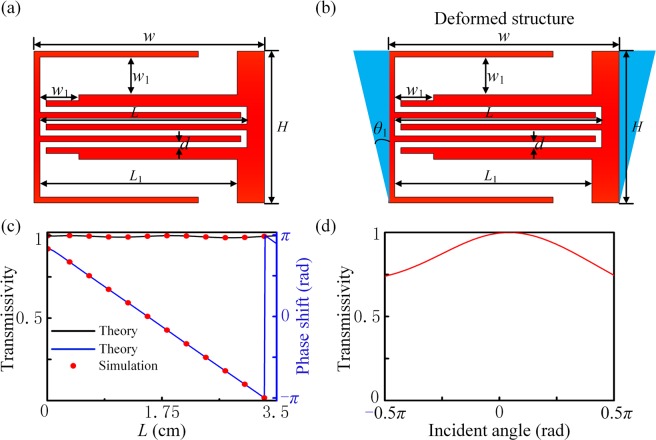
Construction of PCM. (**a**) A labyrinth unit of PCM, with a width *w*(=38 *mm*), height *H*(=25.7 *mm*), thickness of the middle curved pipes *d*(=1 *mm*), length *L*_1_(=26.091 *mm*) and thickness of the two outside pipes *w*_1_(=6.392 *mm*), respectively. **(b)**The deformed trapezoidal labyrinth unit, with the *blue solid triangles* attached on the left and right sides of original structure, is used for circular cloaking with *θ*_1_ = 1.61°. **(c)** Transmissivity and phase shift characteristics of the PCM unit. **(d)** The relation between transmissivity of labyrinth unit and the incident angle.

Here, we use two kinds of labyrinth structures to achieve the PCM and NZIM units, respectively. Figure 3(a) shows a labyrinth unit of the orthotropic PCM. The width and height of the unit is *w* = 38 mm (0.266 *λ*) and *H* = 25.7 mm (0.18 *λ*), respectively. Here, the frequency of the incident waves is 2400 Hz. The unit consists of three curved pipes, in which the two coating pipes on the outside are symmetrical. The thickness of the curved pipes in the middle part is *d* = 1 mm. The effective impedance of the unit is related to the thickness (*w*_1_) and length (*L*_1_) of the two symmetrical layers. In order to match the effective impedance of the unit to that of air, the optimal parameter solution of the unit can be obtained by scanning *w*_1_ and *L*_1_^32^. Here we would like to note that the proposed hybrid metasurface assemble the phase modulation capability of PCM and the equal-phase surface property of NZIM to make the waves propagate in the original direction around the obstacle, which differs from previous composite metasurface in physics principles and functionalities^32^. Here, *w*_1_ and *L*_1_ are set as 6.392 mm and 26.091 mm, respectively. In this case, the effective impedance of the unit matches that of air and then the unit has a high transmittance. It is obvious that the acoustic waves actually propagate along the curved pipes instead of the straight line, and the path length of acoustic waves passing through the unit can be changed by variation of the length *L* for the intermediate curved pipes. When all other parameters of the unit are fixed, the phase shift can be obtained from π to -π by the variation of *L*. Figure 3(c) shows the variation of transmissivity and phase shifts in relationship to length respectively. It is found that the transmissivity is slightly disturbed around 1 with the various *L*. In other words, the effective impedance of unit perfectly matches that of air in the various range of *L* from 0 to 3.5 cm. The phase shift varies linearly from *π* to -*π* with the variation of *L* from 0 to 3.5 cm. For convenience, the phase shifts from *π* to -*π* are discretized into twelve structures with a step of *π*/6, as shown by red dots in Fig. 3(c). The *L*-values of the twelve structures are set as 0.031, 0.342, 0.639, 0.936, 1.233, 1.529, 1.824, 2.123, 2.423, 2.721, 3.012 and 3.311 cm, respectively. The transmissivity of labyrinth unit dependent on the incident angle is shown in Fig. 3(d). It is found that the transmissivity reaches up to 88.93% in average, which maintains over 75% even in the case of grazing incidence. In order to achieve the cloaking of circle obstacle, the unit has to make a transformation. As shown in Fig. 3(b), the outer shape of the unit changes from a rectangle to trapezoid and *θ*_1_ is set as 1.61°, while all the parameters of the internal curved pipe remain unchanged. For example, the length of top side is changed from *w* into *w* + 2*H* tan *θ*_1_ with *θ*_1_ = 1.61°. The transmission and phase shift characteristics of the trapezoidal unit are consistent with that of the rectangle unit.

**Figure 4:**
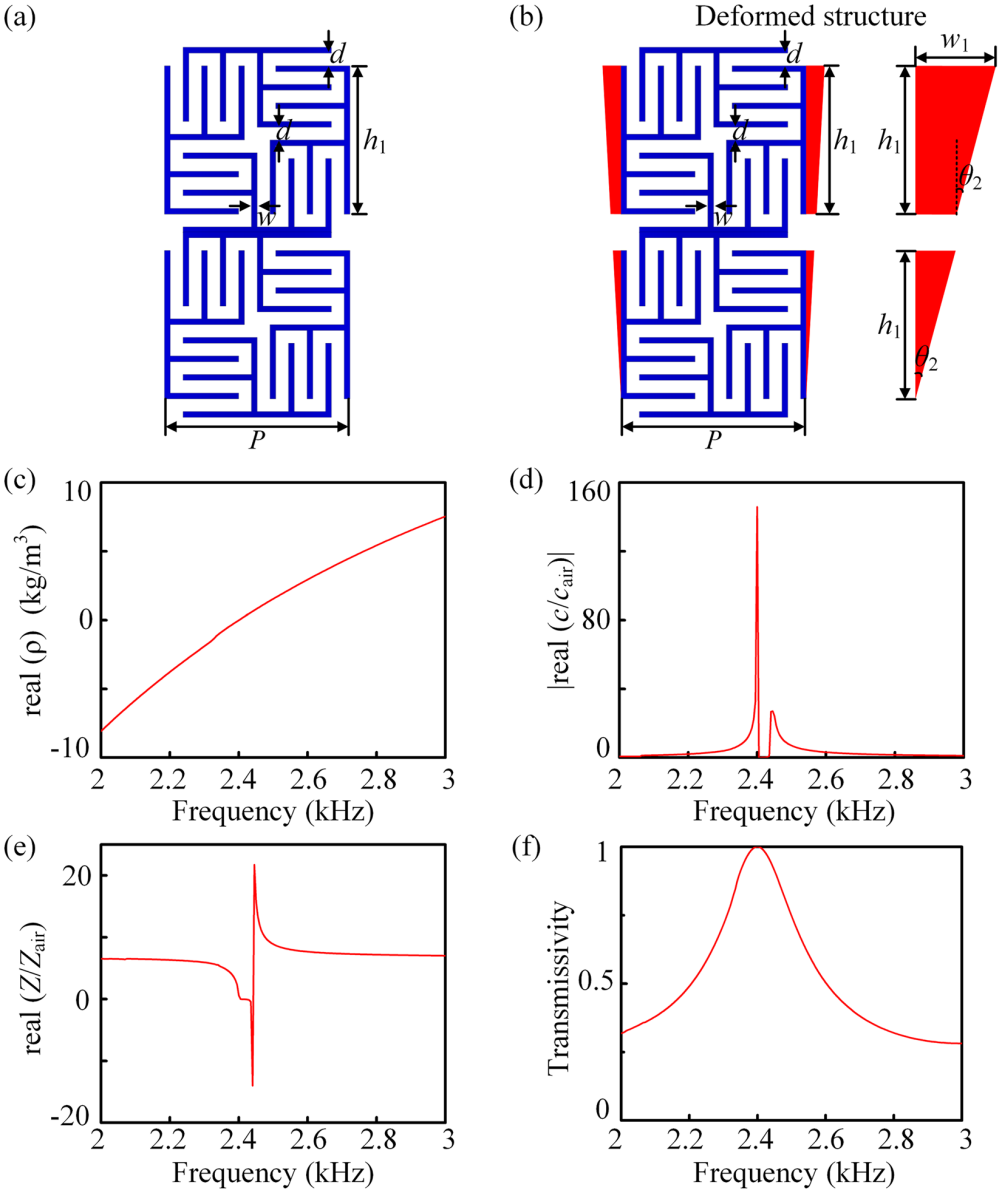
Construction of NZIM. (**a**) A labyrinth unit of NZIM constructed by two layers of the square labyrinth structure, with the parameters *P* = 32 mm, *w* = 1 mm and *d* = 2.2 mm at 2400 Hz. **(b)** The deformed trapezoidal labyrinth unit, with the *red solid trapezoids* and *triangles* attached on the left and right sides of original structure, is used for circular cloaking with *θ*_2_ = 1.5°, with ${h}_{1}=P-2d-2w$ and ${w}_{1}=2({h}_{1}+d+w)\,\tan \,{\theta }_{2}$. Corresponding **(c)** real part of effective density, **(d)** the absolute value of the relative effective velocity, **(e)** real part of normalized effective acoustic impedance, and **(f)** transmissivity of the NZIM unit.

Similarly, a labyrinth unit of isotropic NZIM is constructed by two layers of the square labyrinth structure, as shown Fig. 4(a). When the incident frequency approaches the dipolar resonance, the effective density of the structure is close to zero, then the effective index also approaches zero and the effective acoustic velocity is much greater than the velocity in air. The effective parameters of the structure can be obtained by the effective diffraction index theory^33^. Figure 4(c,d) show that at the resonant frequency 2400 Hz, the real part of the effective density approaches 0 and the absolute value of the real part of acoustic velocity is about 140 times the sound velocity in air. Figure 4(e,f) show that the at the resonant frequency, the normalized effective acoustic impedance equals to 1, indicating that the acoustic waves can totally pass through the structure without any reflections. Thus, the square labyrinth structure can be used to achieve NZIM. Finally, the deformed trapezoidal labyrinth unit can be used in order to make the NZIM conformal with the circular obstacle, with the *red solid trapezoids* and *triangles* attached on the left and right sides as shown in Fig. 4(b). Here, *θ*_2_ = 1.5° and other parameters remain unchanged.

### Acoustic cloaking demonstration

**Figure 5:**
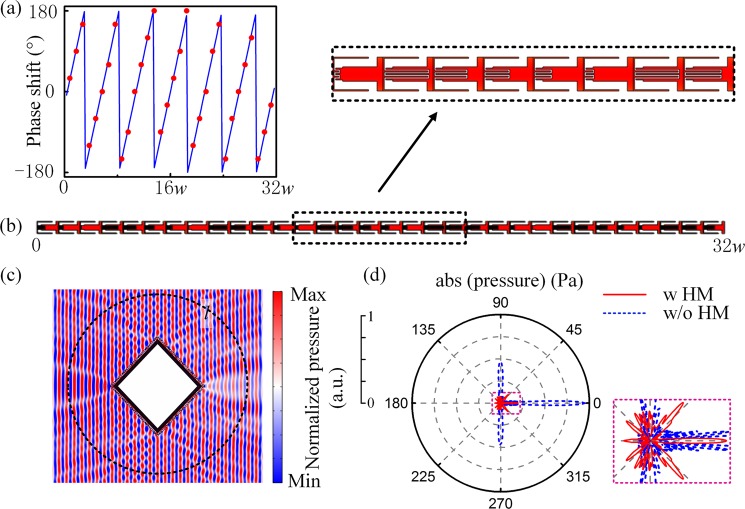
Cloaking of square obstacle. (**a**) Phase shifts of the ideal (*blue curve*) and discretized (*red dots*) square PCM. A quarter of the PCM is illustrated due to the symmetry. (**b**) Corresponding construction details. (**c**) Pressure field distributions when the rigid square obstacle is cloaked by the HM. (**d**) Far-field scattering patterns for the cases of uncloaked (*blue dashed curve*) and cloaked (*red solid curve*) rigid square obstacle.

We use the above two structures to achieve the cloaking of square obstacle and circle obstacle. The size of square obstacle and circle obstacle are the same with that in Fig. 2(b,c). The cloaking of square obstacle is illustrated first. In incident area, the PCM makes the incident plane waves with an incident angle of 45° hitting the NZIM vertically; while in the emergent area, the effect of PCM becomes such that the waves perpendicularly transmit from NZIM can be anomalously refracted at 45°. As shown in Fig. 5(a), the phase profile in one side of square PCM (blue solid curve) can be derived by the generalized laws of refraction^34^, and 32 labyrinth units are used for discretization with the phase shifts induced by each unit represented by red dots. The construction details of 32 labyrinth units are shown in Fig. 5(b). The other three sides of the PCM can be obtained accordingly due to the structure symmetry. Figure 5(c,d) show the pressure field and far-field scattering pattern when the HM is attached to the square obstacle. The incident sound is excited by the background pressure field at *f* = 2400 Hz. It is found that the sound waves mainly propagate in the original incident direction and the scattered waves in other directions can be effectively suppressed. Integrating the scattering intensity along the same circle *l*, the total scattering is decreased from 3.77 W/m^2^ without HM to 0.82 W/m^2^ with HM, which is reduced by 78.2%.

**Figure 6:**
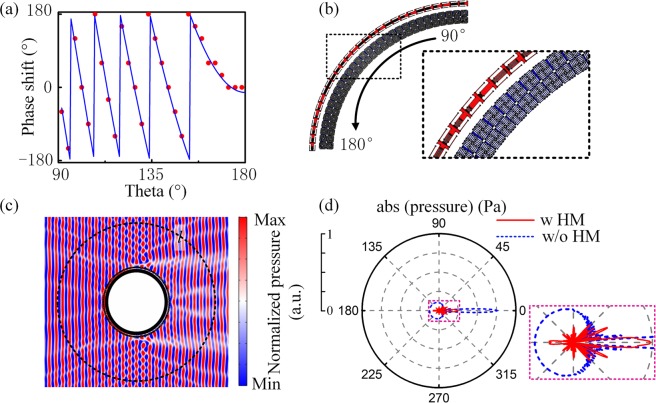
Cloaking of circular obstacle. (**a**) Phase shifts of the ideal (*blue curve*) and discretized (*red dots*) circular PCM. A quarter of the HM is illustrated due to the symmetry. **(b)** Corresponding construction details. **(c)** Pressure field distributions when the rigid circular obstacle is cloaked by the HM. **(d)** Far-field scattering patterns for the cases of uncloaked (*blue dashed curve*) and cloaked (*red solid curve*) rigid circular obstacle.

Figure 6(a,b) show the discrete phase shifts and construction details of a quarter of circular PCM in different azimuth angles. The structures in 0°~90° and 180°~360° can be obtained by mirroring the structures in Fig. 6(b). Similar to the cloaking of square obstacle, the PCM in the incident area is employed to convert the plane waves to cylindrical waves and in the emergent area the PCM actually converts the cylindrical waves from the NZIM back to plane waves. Figure 6(c,d) show the pressure field and far-field scattering pattern when the circular obstacle is covered by the HM, respectively. Here, the frequency of the incident waves is 2395 Hz. The optimal cloaking effect of circle obstacle is found at f = 2395 Hz by frequency scan. This minor change should be ascribed to the deformation of the NZIM structure, which leads to the minor shift in the corresponding frequency of optimal near-zero-refractive-index. PCM is constructed with 112 adjusted structures in Fig. 3(b) and NZIM is constructed with 120 adjusted structures in Fig. 4(b). The total scattering intensity decrease from 2.95 W/m^2^ to 0.78 W/m^2^, which is reduced by 73.6%. Note that although we demonstrate that HM can achieve the cloaking of square and circular obstacles for illustration, this method can be expanded to obstacles in arbitrary shapes.

## Discussion

Ultrathin conformal cloaking of obstacles in arbitrary shapes is demonstrated by the proposed hybrid metasurface, which is composed of coupled PCM and NZIM layers. The ideal acoustic parameters are discretized so that the hybrid metasurface can be constructed by two kinds of labyrinth structures. Different from other methods of acoustic cloaking, the HM is very thin and conformal to the obstacle shapes, without needing any gradient inhomogeneous anisotropic metamaterials of large thickness. The physical hybrid metasurface is wrapped around the obstacles and its thickness is only about 0.62 times the wavelength. Waves can bypass the obstacles and propagate along the original incident direction while scattering waves in other directions are suppressed. These results may promise diverse routes to design the integrated acoustic cloaking based on hybrid metasurfaces with composite functionalities.

## Methods

To demonstrate the acoustic cloaking with the HM, the full wave simulations are performed with the COMSOL Multiphysics based on the finite element method. In the simulations, air (with mass density *ρ* = 1.21 kg/m^3^ and sound velocity *c*_air_ = 343 m/s) and epoxy resin (with mass density *ρ*_1_ = 1050 kg/m^3^ and sound velocity *c*_1_ = 2200 m/s) are used as the background medium and the labyrinth HM structures, respectively. The boundary conditions of obstacle are set as sound hard boundary conditions for intensive sound scattering due to the impedance mismatch. The effect of loss is ignored in the simulations for simplicity (see Supplementary Materials for detailed discussions).

## Supplementary information

Supplementary Materials
