# Supplementary material for: Ultrathin acoustic cloaking by a conformal hybrid metasurface
Source: Sci Rep. 2019 Sep 3;9:12700. doi: 10.1038/s41598-019-49148-3 (PMC6722134; doi:10.1038/s41598-019-49148-3)
Supplement: Supplementary file 1 — Supplementary Materials [file 41598_2019_49148_MOESM1_ESM.pdf]

Supplementary materials to:

Ultrathin acoustic cloaking by a conformal hybrid metasurface

Yihe Wang[1], Ying Cheng[1,2*], Xiaojun Liu[1,2†]

[1]Key Laboratory of Modern Acoustics, Department of Physics and Collaborative Innovation Center of Advanced Microstructures, Nanjing University, Nanjing 210093, China
[2]State Key Laboratory of Acoustics, Institute of Acoustics, Chinese Academy of Sciences, Beijing 100190, China

Correspondence and requests for materials should be addressed to Y.C. (email: chengying@nju.edu.cn) or X.L. (email: liuxiaojun@nju.edu.cn)

The effect of air viscosity

The loss due to thermoviscosity is important when the pipe parameter (the width of pipe) is of the same magnitude as $\sigma_v \approx \sqrt{v/2\pi f}$ and $\sigma_k \approx \sqrt{\alpha/2\pi f}$ [1,2]. Here v is the kinematic viscosity of air, α is the thermal diffusivity, and f is the frequency of the incident wave. σ_v and σ_k are on the order of 10^{-5} in our simulation, which is much smaller than the pipe parameter. Therefore, the viscosity effect is not considered in our model for simplicity.

We further take the air viscosity into consideration, and illustrate the transmissivity and phase shift of PCM unit in Fig. R1. The red and blue curves represent the ideal and viscous properties, respectively. It can be seen from Figs. R1(a) and R1(b) that after including the air viscosity, the transmissivity of PCM unit slightly reduce to around 95% from almost 100% in ideal case, while the phase shift still covers entire 2π in spite of minor change. We also show the influence of air viscosity on the transmittance of NZIM in Fig. R2, in which the maximum transmissivity will reduce to 82% accompany with a frequency shift of 8.75%. We would like to note that the influences of air viscosity were neglected in most previous studies on PCM and NZIM[3-7], and the desired experimental results can also be obtained even when the number of channels is larger and the width of channels is narrower, which induce higher loss than our structure.

Additionally, to further corroborate above results, we also investigate the whole cloaking device taking loss into consideration. The far-field scattering patterns for cloaking of square and circle obstacle are shown in Figs. R3(a) and R3(b), respectively. It is found that the cloaking can still suppress the scattering effectively when considering viscosity, in spite that the cloaking efficiency of square (circle) obstacle reduces from 78.2% (73.6%) in ideal case to 65.4% (60.2%) in viscous case.

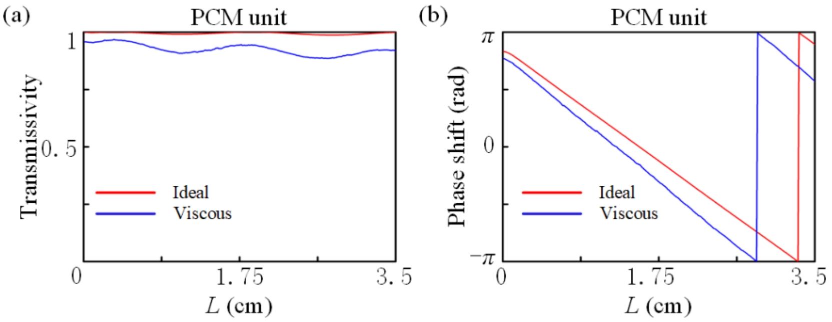

Figure R1: **(a)** Transmissivity and **(b)** phase shift characteristics of the PCM unit. The red and blue curves represent ideal and viscous conditions, respectively.

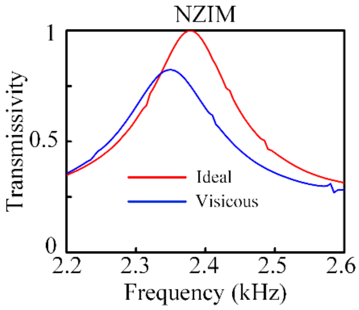

Figure R2: Transmissivity of the NZIM. The red and blue curves represent ideal and viscous conditions, respectively.

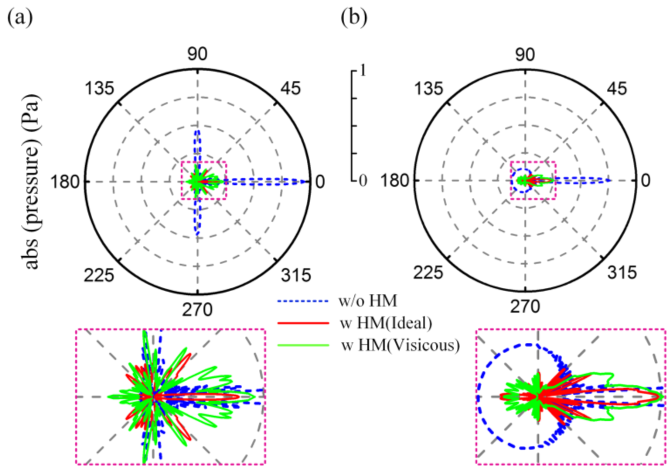

Figure R3: Far-field scattering patterns of the **(a)** square and **(b)** circle obstacle under different conditions. The blue dashed line represents the uncloaked obstacle without HM. The red and green solid lines represent the cloaked obstacle with HM in ideal case and viscous case, respectively.

References

1 Yazaki, T., Tashiro, Y. & Biwa, T. Measurements of sound propagation in narrow tubes. *Proc. R. Soc. A-Math. Phys. Eng. Sci.* **463**, 2855 (2007).

2 Ward, G. P. *et al.* Boundary-Layer Effects on Acoustic Transmission Through Narrow Slit Cavities. *Phys. Rev. Lett.* **115**, 044302 (2015).

3 Li, Y., Liang, B., Gu, Z. M., Zou, X. Y. & Cheng, J. C. Unidirectional acoustic transmission through a prism with near-zero refractive index. *Appl. Phys. Lett.* **103**, 053505 (2013).

4 Zhang, T., Cheng, Y., Yuan, B. G., Guo, J. Z. & Liu, X. J. Compact transformable acoustic logic gates for broadband complex Boolean operations based on density-near-zero metamaterials. *Appl. Phys. Lett.* **108**, 183508 (2016).

5 Chen, D. C., Zhu, X. F., Wei, Q., Wu, D. J. & Liu, X. J. Asymmetric phase modulation of acoustic waves through unidirectional metasurfaces. *Appl. Phys. A-Mater. Sci. Process.* **124**, 13 (2018).

6 Liang, Z. X. & Li, J. S. Extreme Acoustic Metamaterial by Coiling Up Space. *Phys. Rev. Lett.* **108**, 114301 (2012).

7 Shen, C., Xie, Y. B., Li, J. F., Cummer, S. A. & Jing, Y. Asymmetric acoustic transmission through near-zero-index and gradient-index metasurfaces. *Appl. Phys. Lett.* **108**, 223502(2016).
